# Chronic Diarrhea and Weight Loss in a 27-Year-Old: Highlighting Collagenous Gastritis as a Rarely Encountered Entity

**DOI:** 10.3390/diagnostics16091261

**Published:** 2026-04-23

**Authors:** Ádám Ferenczi, Anita Sejben

**Affiliations:** Department of Pathology, University of Szeged, 6725 Szeged, Hungary

**Keywords:** collagenous gastritis

## Abstract

A 27-year-old male presented with chronic diarrhea, bloating, and abdominal pain since age 13. Initially attributed to lactose intolerance, treated with dairy-free diet, symptoms persisted despite negative workup—normal celiac serology, stool studies, and abdominal ultrasound. Recent symptoms included severe diarrhea, fatigue, weakness, 8 kg weight loss, hair loss, elevated IgE and fecal calprotectin. Gastroscopy showed flattened, granular gastric mucosa with focal hyperemia in the antrum and greater curvature. Histology revealed severe chronic inactive H. pylori-negative gastritis with a prominent subepithelial collagen band (verified by Crossmon’s trichrome), confirming collagenous gastritis—a rare entity first described in 1989. The condition has a slight female predominance and bimodal age peaks (adolescence and >60 years). Symptoms are nonspecific, including abdominal pain, diarrhea, weight loss and anemia. Pediatric cases often feature nodular mucosa and anemia; adults more commonly present with watery diarrhea, sometimes linked to collagenous colitis. Diagnosis requires histological features including patchy subepithelial collagen band ≥ 10 μm thick, lymphocytic or eosinophilic infiltration of the lamina propria, epithelial changes and entrapped capillaries. Patterns include atrophic, lymphocytic-like, and eosinophil-rich. Crossmon’s or Masson’s trichrome, Congo red, and tenascin immunohistochemistry aid in proving collagen and excluding amyloidosis. Treatment is mainly symptomatic or with proton pump inhibitors; corticosteroids may be effective in refractory cases.

**Figure 1:**
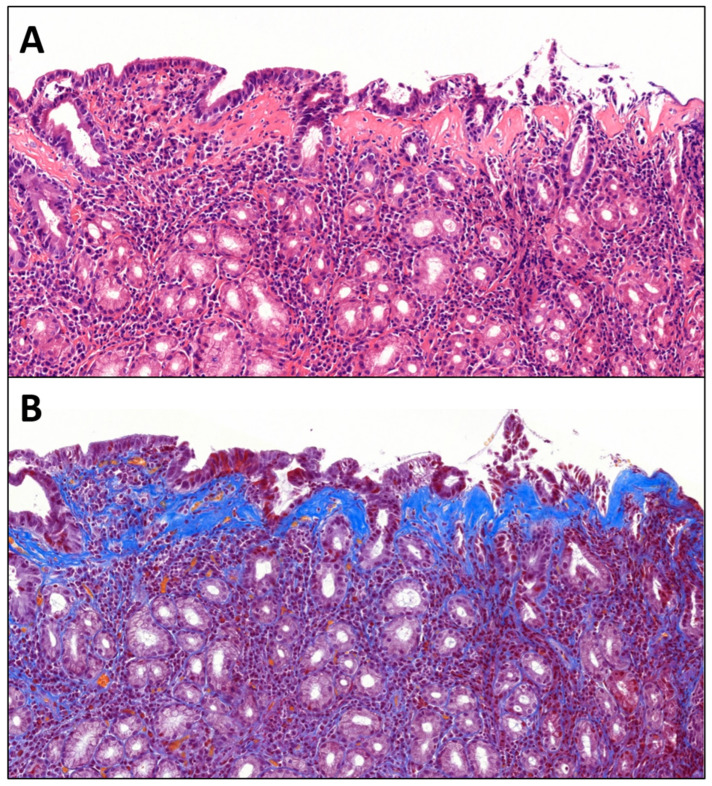
A 27-year-old male presented for gastroscopy and colonoscopy due to long-standing, unexplained gastrointestinal symptoms, including chronic diarrhea. The patient first experienced diarrhea, bloating, and abdominal pain at 13 years of age, at which time pediatric evaluation confirmed lactose intolerance. Episodes of diarrhea appeared to be exacerbated by anxiety, raising suspicion for irritable bowel syndrome; however, pediatric psychiatric evaluation revealed no abnormalities. A dairy-free diet was subsequently implemented, but symptoms persisted. Laboratory evaluation demonstrated normal serum tissue transglutaminase (TTG) levels, negative fecal bacteriology and blood tests, and abdominal ultrasound findings were unremarkable. In the year preceding the current evaluation, the patient experienced an episode of diffuse abdominal pain accompanied by profuse diarrhea, fatigue, and weakness, and was not taking any kind of medications. Fecal bacteriology and parasite tests were negative, while fecal occult blood testing was negative on two occasions and positive on one. Serum TTG levels remained within the normal range (reference: <10 U/mL), while immunoglobulin E (IgE) was mildly elevated (768 IU/L, reference: <100 IU/L) and fecal calprotectin was slightly above normal (110 μm/g, reference: <100 μm/g). Serial measurements of hemoglobin, serum iron, transferrin saturation, and ferritin remained within normal ranges, with no evidence of iron deficiency anemia. At the most recent follow-up, diarrhea persisted, and the patient had experienced an 8 kg weight loss over the preceding year. He reported a vigorous exercise routine and hair loss. Evaluation by ENT specialists revealed no source of chronic inflammation. The patient was currently using minoxidil and high-protein dietary supplements. Gastroscopy and colonoscopy resulted in limited biopsy specimens; thus, re-biopsy was recommended. Most recent gastroscopy reported flattened gastric mucosa along the greater curvature and antrum, finely granular appearance and focal hyperemia. Final biopsy confirmed severe, chronic, inactive, Helicobacter pylori negative gastritis present in both the gastric corpus and antrum, without signs of metaplasia and with a prominent, subepithelial collagen band visible (15–20 µm). An elevated number of lymphocytes and plasma cells could be observed in the lamina propria (**A**). Our case did not demonstrate entrapped capillaries, epithelial detachment or neutrophil granulocytic infiltrate indicative of active inflammation. Crossmon’s trichrome staining confirmed the collagenous composition of the subepithelial band, supporting the diagnosis of collagenous gastritis (**B**). Congo red staining was negative upon evaluation. The prior colonoscopy demonstrated histologically unremarkable colonic mucosa, with no evidence of chronic or active inflammation. Additionally, there was no significant intraepithelial lymphocytosis or subepithelial collagen deposition, thereby excluding features consistent with microscopic colitis. The patient received corticosteroid therapy and is currently asymptomatic. Collagenous gastritis was first reported by Colletti et al. in 1989, describing it as a patchy gastritis with a striking, thick, subepithelial band of collagen that does not extend into the deeper portions of the lamina propria [1]. The disease is hypothesized to be a part of the collagenous gastroenteropathies spectrum, alongside collagenous sprue and collagenous colitis [2,3]. Based on the literature, the incidence is approximately 13/100,000 upper endoscopies, a slight female predominance can be observed, and a bimodal age distribution can be seen with the first peak between ages 10–19 and the second above 60 years. Symptoms are generally nonspecific and persistent, most common manifestations include abdominal pain, diarrhea, iron deficiency anemia, and weight loss [2,3,4,5,6,7]. Clinically, two subgroups of collagenous gastritis have been proposed: children, who typically present with a nodular gastroscopic appearance and iron deficiency anemia, and adults, who more commonly exhibit chronic watery diarrhea often associated with collagenous colitis [8]. However, not all patients conform to these categories [9]. Endoscopic appearance is often described as nodular, however, not further specified [2,3,5,10]. Histology remains the gold standard of diagnosis, with a subepithelial, patchy band of collagen deposition at least 10 μm thick and an associated lymphocytic or eosinophilic infiltrate of the lamina propria, as diagnostic criteria. Epithelial flattening or detachment, as well as entrapped capillaries are also characteristic findings [2,4,5,9,11,12,13,14]. Due to its rarity, the inflammatory patterns observed in collagenous gastritis have not been comprehensively characterized. Arnason et al. described three distinct patterns, including an atrophic, a lymphocytic gastritis–like, and an eosinophil-rich pattern. Among these, the atrophic pattern appears to be the most common, likely reflecting its association with chronic inflammation. In contrast, the eosinophil-rich pattern is considered the most under-recognized and underreported in the literature, which may be attributable to the physiological abundance of eosinophilic granulocytes in the gastrointestinal tract and the lack of well-defined diagnostic thresholds [2,9]. Accompanying intraepithelial lymphocytes have been observed in few of the reported cases, and while not included in the diagnostic criteria, their proportion must not reach the diagnostic threshold for lymphocytic gastritis [2]. Varying degrees of chronic infiltrate can often be seen; while signs of activity may be present, however, it is not part of the diagnostic requirements [4]. Crossmon’s or Masson’s trichrome and Congo red stains, as well as tenascin immunohistochemistry may help with diagnosis and elimination of amyloidosis from the differential diagnostic palette [2,9,15]. Further differential diagnostic considerations include autoimmune gastritis-, or radiation therapy-associated fibrosis, characterized by diffuse collagen deposition not limited to a subepithelial band, as well as scleroderma, in which the entire thickness of the mucosa and possibly the deeper layers are also affected. Tangential sectioning may also lead to a misperception of the thickness of the subepithelial collagen [16]. Collagenous gastritis can exhibit features resembling eosinophilic gastritis; however, a count of ≥30 eosinophils per high-power field supports the diagnosis of eosinophilic gastritis [17]. Treatment most commonly includes symptomatic management or proton pump inhibitors and in therapy resistant cases good response has been described to oral or topical corticosteroids [5,7]. This case may contribute to awareness to under-recognition due to the rarity of collagenous gastritis in routine practice, including the patient’s nonspecific clinical features (chronic watery diarrhea, weight loss, and only mildly abnormal laboratory findings) and the potential for overlap with more common conditions in the differential diagnosis.

## Data Availability

The original contributions presented in this study are included in the article. Further inquiries can be directed to the corresponding author.

## Abbreviations

The following abbreviations are used in this manuscript:

ENT	Ears, Nose, and Throat
IgE	Immunoglobulin E
TTG	Serum tissue transglutaminase
